# Characterization of Phase Change Materials Fabricated with Cross-Linked Graphene Aerogels

**DOI:** 10.3390/gels8090572

**Published:** 2022-09-08

**Authors:** Chengbin Yu, Young Seok Song

**Affiliations:** 1 Department of Materials Science and Engineering, Research Institute of Advanced Materials (RIAM), Seoul National University, Seoul 08826, Korea; 2 Department of Fiber Convergence Materials Engineering, Dankook University, Yongin-si 16890, Korea

**Keywords:** graphene aerogel, phase change material, cross-linked, thermal energy storage

## Abstract

3D porous graphene aerogel exhibits a high surface area which can hold plenty of pure phase change material (PCM) into the internal space. In order to maintain the flexibility of PCM without volume shrinkage under the external force, cross-linked graphene aerogel was prepared by the cysteamine vapor method. The cross-linked graphene aerogel had a high stress–strain durability and chemical stability for infiltrating PCM to produce a form-stable PCM composite. The latent heat of PCM is one of the elements to estimate the capacity of PCM thermal energy storage (TES) during the phase transition process. The cross-linked graphene aerogel-supported PCM composite showed a great TES to be utilized in thermal-to-electrical energy harvesting. The cross-linked graphene aerogel also had an excellent mechanical property of preventing damage at a high temperature.

## 1. Introduction

3D porous technology is one of the hot issues in various industrial applications due to its light weight, high porosity, and low cost. The porous materials exhibit internal structures that can infiltrate plenty of solid–liquid working materials into the internal space [1,2]. That is to say, the supporting materials with high porosity can serve as a container with a small weight fraction [3,4]. To develop a porous supporting material for practical applications, synthesizing a foam structure is needed to match working materials [5,6]. In comparison to a typical foam, the 3D aerogel shows relatively low density, i.e., lots of pores in the internal structure [7,8]. The graphene aerogel is appropriate for fabricating polymer composite due to its high chemical stability and mechanical property [9,10]. In many cases, graphene oxide (GO) is synthesized and dispersed in distilled water to manufacture a 3D porous GO aerogel [11,12]. The functional groups of GO entail hydrogen bonding to reinforce the connection with graphene skeletons and produce a stable aerogel structure [13,14]. After reducing the functional groups, graphene aerogel is fabricated as supporting material.

However, one of the recent problems in the graphene aerogel is the volume shrinkage while infiltrating a polymer into the internal space [15,16]. The capillary force in the aerogel pores leads to the deformation of porous skeletons, which affects the internal porosity [17]. In order to keep plenty of working materials for a stable 3D graphene aerogel, the supporting material needs to increase its flexibility and mechanical property [18,19]. One of the synthesis methods for a modified graphene aerogel is to mix flexible materials with polydimethylsiloxane (PDMS) [20,21]. The PDMS embedded graphene aerogel has excellent flexibility and recovery feature to the initial state after removing the external force [22]. Thus, PDMS-embedded modified graphene aerogel is subsequently utilized to reduce the volume shrinkage effectively during the infiltrating process [23,24]. On the other hand, thermal energy storage (TES) and thermoelectric energy harvesting can resolve the energy crisis and environmental pollution [25,26]. Phase change material (PCM) is the most popular for thermal energy applications because of absorbing or releasing a large amount of heat during the phase transition process [27,28].

It is necessary to utilize graphene aerogel for fabricating form-stable PCM composite due to the leakage problem under the solid–liquid melting process [29,30]. The melted PCM shows weight loss and deformation, which cannot materialize a high TES ability [31,32]. In order to sustain an intrinsic TES capacity without any leakage, 3D porous graphene aerogel needs to fabricate a PCM composite with high form stability [33,34]. The PCM composite shows a high TES capacity to store or release thermal energy and can be connected to a thermoelectric power generator (PEG) for thermoelectric energy harvesting [35]. The modified graphene aerogel reduces volume shrinkage effectively and keeps the high porosity to infiltrate plenty of pure PCM [36]. The modified graphene aerogel-supported PCM composite has a decrease in its mechanical property when the pure internal PCM is changed to a liquid state [37]. It is easy to occur a leakage problem under the existence of external force, and increasing the mechanical property of modified graphene aerogel is vital to keep the initial shape under the external force [38,39]. It is reported that the cross-linked modified graphene aerogel uses cysteamine to have a reaction with GO functional groups [40]. The cross-linked graphene aerogel is an advanced supporting material to infiltrate pure PCM without any volume shrinkage and sustain the solid state of PCM composite during the phase transition process.

In this study, the importance of modified graphene aerogel shows not only the reduction of volume shrinkage but also the prevention of the leakage problem under external force. There are conventional graphene aerogel, PDMS embedded graphene aerogel, and cross-linked graphene aerogel with some typical morphologies, and cross-linked graphene aerogel supported PCM composite shows excellent form-stability under the external force at a high temperature. For the microencapsulation method of fabricating form-stable PCM composite, the replacement of supporting shell material causes a decrease in pure PCM weight fraction, which affects the TES capacity during the phase transition process. The graphene aerogel-supported PCM composite has a high PCM weight fraction and keeps a high TES capacity for utilizing stored energy sufficiently. It indicates that cross-linked graphene aerogel is an optimized supporting material and can be employed to fabricate tremendous form-stable PCM composites.

## 2. Results and Discussion

### 2.1. Characteristics of Graphene Aerogels

**Figure 1:**
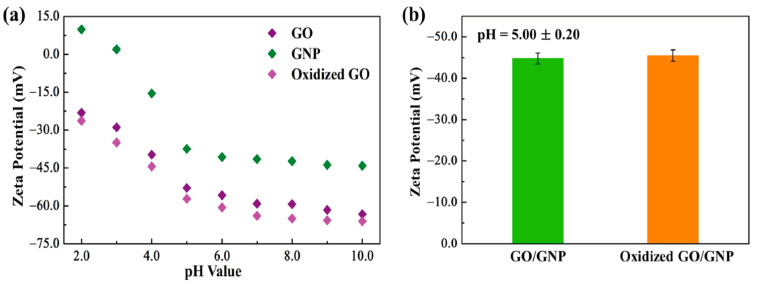
(**a**) Zeta potential results of graphene oxide (GO), graphene nano-platelet (GNP), and KMnO_4_ oxidized GO. (**b**) The mixture of GO/GNP and oxidized GO/GNP solutions.

**Figure 2:**
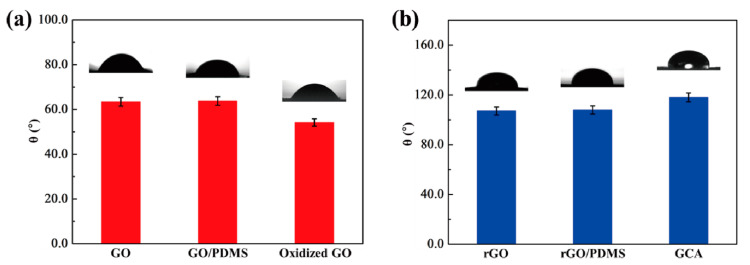
Contact angles of (**a**) GO, GO/PDMS, and oxidized GO, (**b**) rGO, rGO/PDMS, and GCA.

**Figure 3:**
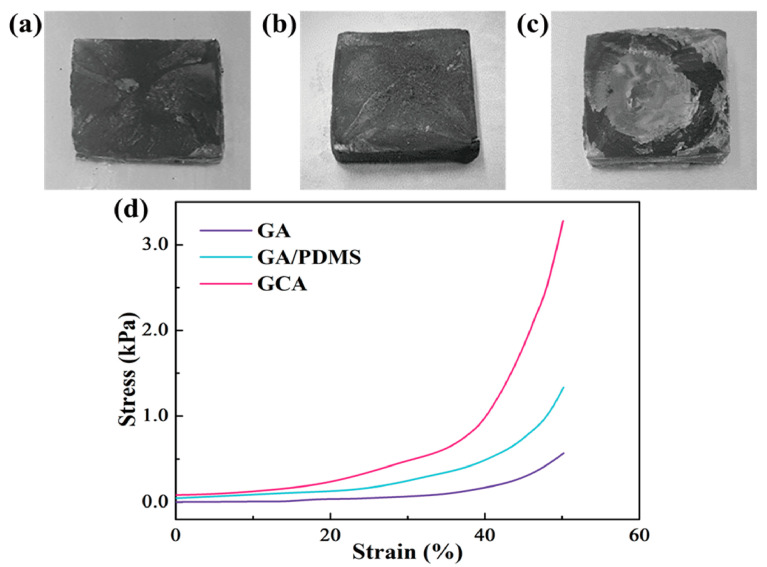
Photographic images of (**a**) graphene aerogel (GA), (**b**) GA/PDMS, and (**c**) GCA. (**d**) Stress–strain curves of the graphene aerogels.

**Figure 4:**
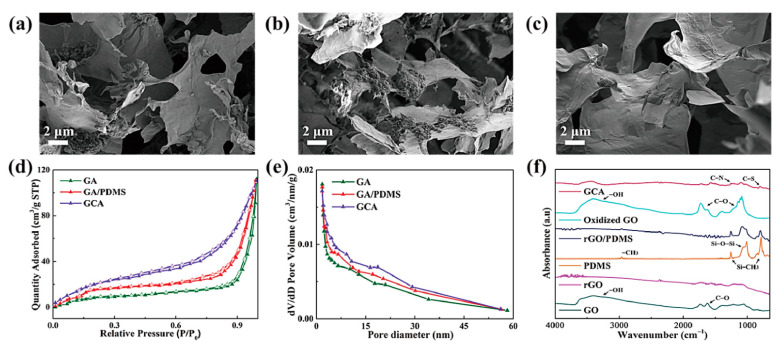
SEM images of (**a**) GA, (**b**) GA/PDMS, and (**c**) GCA. Graphene aerogels BET measurement of (**d**) nitrogen adsorption–desorption peaks, and (**e**) pore size distribution peaks. (**f**) FTIR peaks of the graphene supporting materials.

In order to confirm the morphologies of graphene aerogels, the dispersion property in the DI water was analyzed. The result of zeta potential is shown in Figure 1. Both the original graphene oxide (GO) and KMnO_4_ oxidized GO had high values in the range of 4–10 pH values, as shown in Figure 1a. The blends of GO/graphene nano-platelet (GNP) presented high dispersion ability. It indicates that GO can be used together with GNP to fabricate aerogel. Figure 1b shows that the GO/GNP was dispersed well in the DI water, which is necessary to produce the 3D porous aerogel structures. The modified graphene aerogels require hydrophilic properties. Figure 2 shows the results of contact angle. All of the GO aerogel sheets exhibited lower contact angles than the reduced graphene oxide (rGO) sheets due to the functional groups of graphene skeletons. The cross-linked graphene aerogel (GCA) had the best hydrophobic property, which can facilitate the infusion of melted polymers. The images of the graphene aerogels are shown in Figure 3a–c. The hydrazine- and cysteamine-treated GO aerogels turned black and were reduced to the graphene aerogels. The PDMS embedded graphene aerogel (GA/PDMS) and GCA had better mechanical properties than the original graphene aerogel (GA), as given in Figure 3d. The stress–strain peaks of GA merely endured 0.5 kPa, while the flexible GA/PDMS showed maximum stress of 1.2 kPa. The cross-linked chemical bonding can resist the external force up to 3.3 kPa, which can lead to the utilization as an advanced supporting material. Figure 4 shows the internal structure of the graphene aerogels, figures of nitrogen adsorption–desorption isotherms by BET measurement, and the FTIR result. The modified graphene aerogels had similar pore structures to conventional GA and exhibited high surface areas from Figure 4a–c. The weight of graphene aerogels and BET isothermal nitrogen adsorption–desorption results are listed in Table 1. The internal structures of graphene aerogels were measured by type III isotherms and the multilayer adsorption on the microporous structure under the 0.95 P/P_0_ high-pressure region (Figure 4d). Two kinds of modified GAs can exhibit high specific surface area and high internal volume of porosities. It suggests that the cross-linked GCA has a high internal space to infiltrate plenty of pure PCM without any leakage. Figure 4e shows the pore diameter size distribution, and most of the pore size is close to the range of 0–15 nm under the nitrogen treatment, and it indicates the graphene aerogels had stable microporous structures. The peaks of GA pore size were similar and merely a little bit different due to the change in internal pore structures. The nitrogen adsorbed all internal pores of GA, including tiny gaps between graphene skeletons. Thus, the results of pore size were nanoscale and concentrated on the range of diameter below 15 nm. It indicated that the GA showed a micro-porous internal volume and infiltrated plenty of pure PCM. The cysteamine-reacted GO showed C-N and C-S bonding at 1250 cm^−1^ and 750 cm^−1^. It was demonstrated that the cross-linked structures were generated in the GCA skeletons (Figure 4f). The comparison of graphene aerogels shows that GCA can infiltrate plenty of polymers into the internal space and be used as a container with high stability.

### 2.2. Properties of PCM Composites

**Figure 5:**
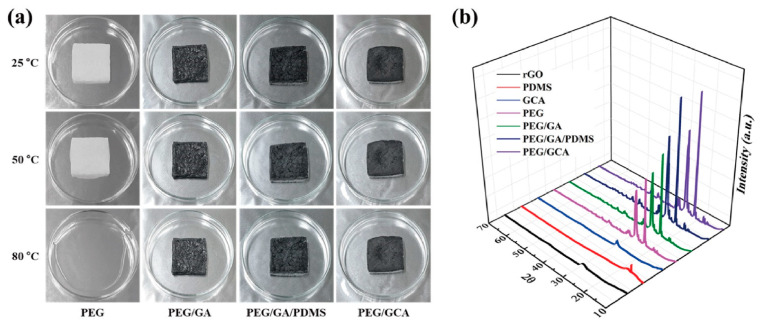
(**a**) Form-stable photographic images of pure PEG and PEG composites. (**b**) XRD peaks of supporting materials and PEG composites.

**Figure 6:**
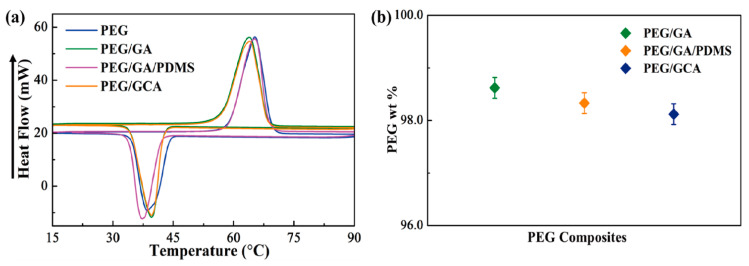
DSC results of (**a**) the pure PEG and PEG composites. (**b**) PEG weight fractions of the PEG composites.

The fabricated graphene aerogels exhibited high porosities and appropriate mechanical properties. The form-stable test indicates that the graphene aerogels can act as excellent supporting materials to prevent the leakage problem (Figure 5a). In order to observe the form stability, the pure PEG and PEG composites were placed on the hot plate, increasing the temperature from 25 °C to 80 °C. When the temperature was increased up to 50 °C, the pure PEG showed a little leakage and fully melted to the liquid state at 80 °C. On the contrary, the PEG composites maintained their initial shapes during the melting process without any leakage. The supporting materials and PEG composites were employed for the XRD test, as shown in Figure 5b. In the case of the pure PEG and PEG composites, two intrinsic peaks at 20–25° were observed. The XRD results indicated high chemical stability between supporting materials and pure PEG. In order to observe the thermal energy storage (TES) capacity of PEG composites, the DSC measurement was conducted. Figure 6a shows the DSC results during the heating and cooling processes. The peaks of the PEG composites were shifted a little bit, and similar latent heat (∆H) was obtained, as given in Table 2. The PEG/GCA composite showed latent heats of 178.27 J/g and 159.06 J/g during the phase transition process, which were close to those of the pure PEG and PEG/GA composite. It can be inferred from the results of melting temperature (T_m_) that the PEG can be melted at 80 °C. Graphene aerogels need high mechanical properties to sustain the melted PEG in the internal pores. The pure PEG weight fractions of each PEG composite were presented in Figure 6b. The modified graphene aerogels containing PDMS and cysteamine chemical bonding showed weight fractions of 98.33% and 98.15%, respectively. The pure PEG had a weight fraction of 98.62% in the PEG/GA composite. All the PEG composites had a large amount of working material to absorb and release thermal energy during the phase transition process.

### 2.3. Form-Stability of PCM Composites

**Figure 7:**
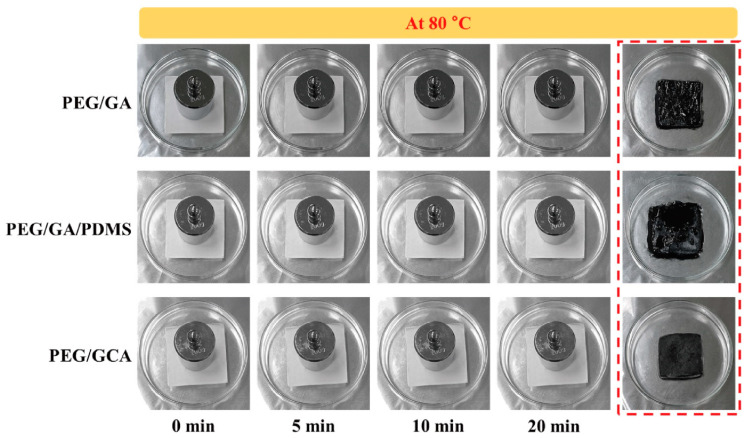
Form-stable photographic images of the PEG composites under the external force at 80 °C. The PEG/GA and PEG/GA/PDMS show the damage of structures after removing the weight. The PEG/GCA keeps initial state without any leakage due to the high mechanical property of GCA.

**Figure 8:**
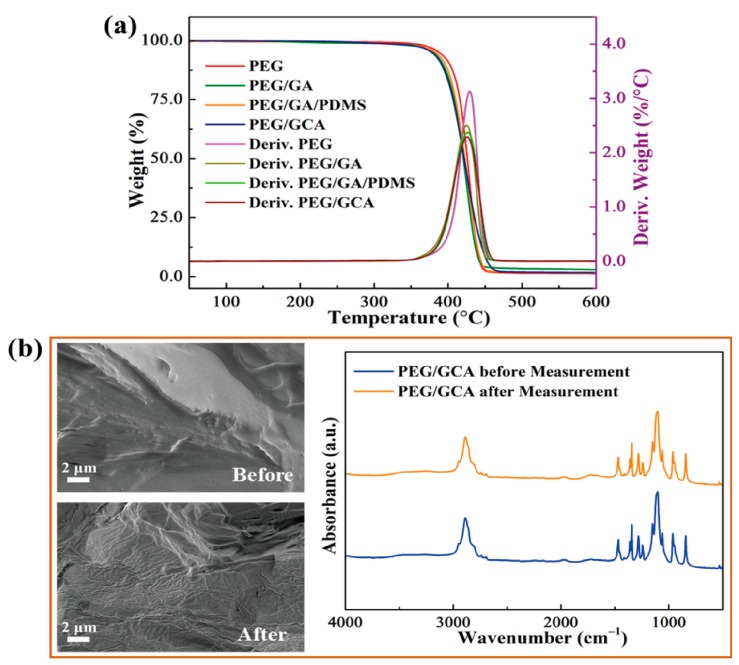
(**a**) TGA peaks of the pure PEG and PEG composites. (**b**) SEM and FTIR results of the PEG/GCA composite after TGA measurement.

It was demonstrated that the graphene aerogel-supported PEG composites had excellent form stabilities during the melting process. In order to observe the durability of external force at a high temperature, all of the samples were placed on the hot plate under 80 °C. An external force of 2 N was applied, and the resulting deformation of the PEG composites was presented in Figure 7. After removing the external force, the PEG/GA and PEG/GA/PDMS composites showed serious damage and leakage problem due to their weak mechanical properties. The cross-linked GCA exhibited high durability to maintain the initial solid state perfectly. It is shown that the GCA-supported PEG composite can absorb and release thermal energy without any leakage under the impact of external force. In order to confirm the thermal stability during the range of temperature variation, the TGA results were illustrated in Figure 8a. The pure PEG and PEG composites exhibited high thermal stabilities under the phase transition field as listed in Table 3. Figure 8b shows the SEM images and FTIR peaks of the PEG/GCA composite after the TGA measurement. The PEG was fully infiltrated into the GCA internal pores. After the TGA measurement, the PEG/GCA still kept excellent thermal and chemical stabilities at high temperatures.

## 3. Conclusions

In this work, we fabricated the graphene aerogel, PDMS embedded graphene aerogel, and cross-linked graphene aerogel (GCA) for 3D porous supporting materials. The modified graphene aerogel, especially the cross-linked GCA sheet, showed a high value of contact angle to infiltrate pure PCM effectively. All the graphene aerogel exhibited nearly 370 of their specific surface area under the BET measurement. High dimensional stability and durability of graphene aerogels are required to infiltrate a large amount of pure PCM, which is over 98%, into the internal space without any volume shrinkage. The modified graphene aerogels exhibited great flexibility to reduce the volume shrinkage effectively. Since the form-stability of PCM composites at high temperatures is essential for applications of PCM. The mechanical properties of the graphene aerogels supported PCM composites were analyzed under the 2N external force during the phase transition process. The cross-linked graphene aerogel could retain the PCM composite without any leakage, even at 80 °C. The cross-linked porous aerogel showed a large potential as a light-weight container for aerospace applications.

## 4. Materials and Methods

### 4.1. Materials

Polyethylene glycol (PEG Mn = 6000; Avention Corporation, Incheon, Korea) was selected as a phase change material (PCM). Graphene nano-platelet (GNP, C grade), Polydimethylsiloxane (PDMS), nitric acid (HNO_3_), sulfuric acid (H_2_SO_4_), potassium permanganate (KMnO_4_), perchloric acid (HClO_4_), and citric acid were purchased from Sigma-Aldrich (St. Louis, MO, USA).

### 4.2. Fabrication of Graphene Aerogels and PCM Composites

**Figure 9:**
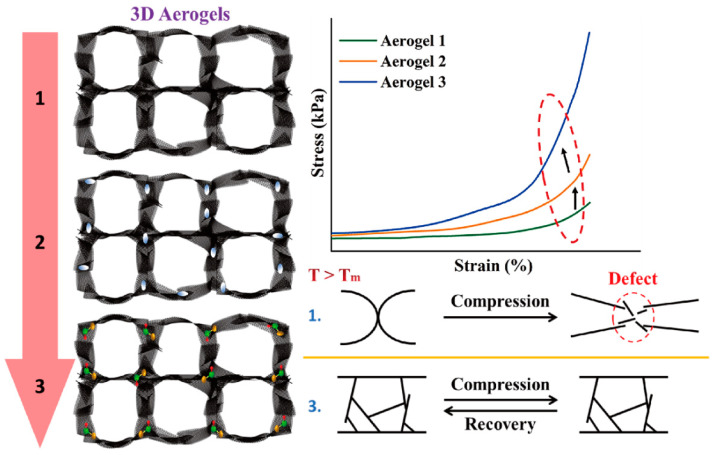
Schematic of the 3D porous graphene aerogels with different internal structures. The cross-linked graphene aerogel has the best mechanical properties to reduce the volume shrinkage effectively. The final PCM composite can sustain the initial solid state without any damage under the external force at a high temperature.

The first step of fabricating porous graphene aerogel was synthesizing graphene oxide (GO). The preparation of GO was followed by the modified Hummers’ method after grinding the graphite [41,42]. The synthesized GO powder was dispersed into the DI water by ultrasonication, and the graphene nano-platelet (GNP) was put into the GO solution to produce GO/GNP mixture. The 4 cm × 4 cm mold was utilized to fill into the GO/GNP mixture and obtain the GO aerogel by freeze-drying method for 72 h. The conventional graphene aerogel (GA) was fabricated using the hydrazine vapor method to reduce the GO functional groups [43]. The modified graphene aerogel was mentioned due to the volume shrinkage during the infiltration process; polydimethylsiloxane (PDMS) was diluted with n-Hexane and sprayed the PDMS solution into the GA internal skeletons. The PDMS embedded GA was then placed into the vacuum chamber at 80 °C for over 2 h to remove the n-Hexane [44]. The PDMS embedded GA was labeled as GA/PDMS in this study. In order to increase the mechanical property, cross-linked supporting material was synthesized by using nitric acid (HNO_3_) and potassium permanganate (KMnO_4_)/perchloric acid (HClO_4_) solution to oxidize initial GO powders. After removing the excess KMnO_4_ by adding citric acid, the oxidized GO was purified by centrifugation and freeze-drying process. The oxidized GO aerogel was obtained by mixing with GNP, evaporating solvent, and utilizing the cysteamine vapor method to cause a chemical reaction with GO functional groups [45,46]. As a result, cross-linked graphene aerogel was synthesized successfully. It was labeled GCA in the current study. The increase in mechanical property and flexibility is briefly shown in Figure 9. The 3D porous graphene aerogels can sustain their high porosities to keep the pure PCM high weight fraction with form stability. The polyethylene glycol (PEG) was melted and put into the different graphene aerogels [47].

### 4.3. Characterizations

The graphene oxide (GO) and Graphene nano-platelet (GNP) dispersion abilities were measured by a Zetasizer (Malvern, UK). The fabricated graphene sheets were put in the contact angle goniometer (Attension Theta Lite, Biolin Scientific, Gothenburg, Sweden) to obtain hydrophilic properties. The maximum stress–strain durability of graphene aerogels was observed by a Universal Testing Machine (UTM, Instron, Norwood, MA, USA). Field-emission scanning electron microscopy (FE-SEM, Merlin compact, ZEISS, Oberkochen, Germany) was used to measure the internal surface structures of graphene aerogels and PCM composites under a 5 kV accelerating voltage. A Brunauer–Emmett–Teller (BET) analyzer (Micromeritics ASAP 2020, USA) was provided to measure the graphene aerogel internal structures. The intrinsic peaks were confirmed using Fourier-transform infrared spectroscopy (FTIR, Varian 660, UT, USA) and X-ray diffraction (XRD, New D8, Bruker, Billerica, MA, USA) from 10° to 70° with 3°/min rate (2θ). The phase transition field and latent heat (∆H) of PCM composites were obtained using differential scanning calorimetry (DSC 4000, PerkinElmer, Waltham, MA, USA) under the range of 15 °C to 90 °C with 10 °C/min. The thermal stability was confirmed by using a thermal gravimetric analyzer (TGA, TA Instruments, New Castle, DE, USA).

## Figures and Tables

**Table 1:** The characterization of the graphene aerogels.

Samples	GA	GA/PDMS	GCA
Weight (g)	0.09 ± 0.10	0.15 ± 0.10	0.17 ± 0.10
Specific Surface Area (m^2^/g)	370.51 ± 0.10	370.24 ± 0.10	369.58 ± 0.10
Average pore size by N_2_ (nm)	12.64 ± 0.10	12.49 ± 0.10	12.08 ± 0.10
Porosity (%)	98.39 ± 0.10	98.31 ± 0.10	97.87 ± 0.10
Total Pore Volume (cm^3^/g)	56.28 ± 0.10	49.90 ± 0.10	45.05 ± 0.10

**Table 2:** Thermal characteristics of the PEG and PEG composites.

Samples	T_mp_ (°C)	ΔH_m_ (J/g)	T_cp_ (°C)	ΔH_c_ (J/g)
PEG	65.27	181.63	38.46	161.98
PEG/GA	63.38	179.23	39.28	159.80
PEG/GA/PDMS	65.22	178.61	37.35	159.32
PEG/GCA	63.37	178.27	39.21	159.06

**Table 3:** TGA characteristics of the pure PEG and PEG composites under N_2_ atmosphere.

Samples	Onset (°C)	Peak (°C)	Endset (°C)	Residual Mass (%)
PEG	388.26	429.11	448.54	1.26
PEG/GA	381.64	425.74	446.02	2.70
PEG/GA/PDMS	384.71	427.86	449.86	2.71
PEG/GCA	381.42	426.52	462.27	2.84

## Data Availability

Data will be made available upon reasonable request to the corresponding author.
