# Correction: Yu, C.; Song, Y.S. Characterization of Phase Change Materials Fabricated with Cross-Linked Graphene Aerogels. *Gels* 2022, *8*, 572

**DOI:** 10.3390/gels12010059

**Published:** 2026-01-08

**Authors:** Chengbin Yu, Young Seok Song

**Affiliations:** 1 Department of Materials Science and Engineering, Research Institute of Advanced Materials (RIAM), Seoul National University, Seoul 08826, Republic of Korea; ycb0107@snu.ac.kr; 2 Department of Fiber Convergence Materials Engineering, Dankook University, Yongin-si 16890, Republic of Korea

## Error in Figure

In the original publication [1], there was a missing self-citation in Figure 3 and a mistake in Figure 4 as published. The corrected Figure 4 is shown below.

**Figure 3:**
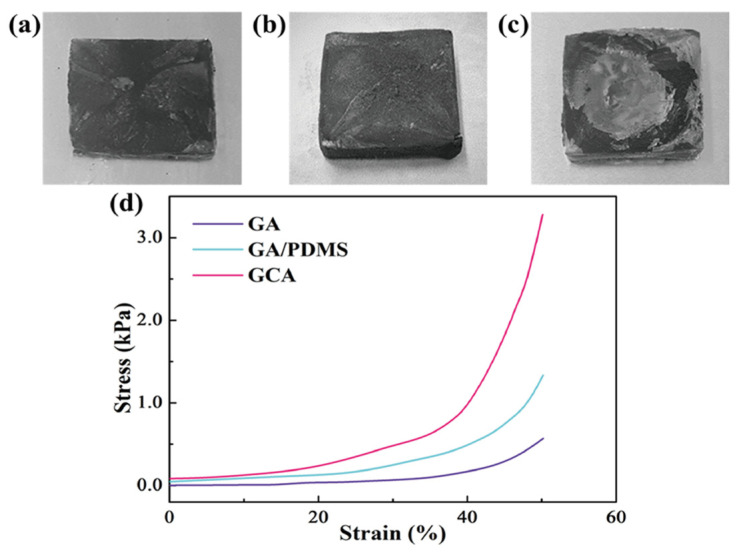
Photographic images of (**a**) graphene aerogel (GA), (**b**) GA/PDMS, and (**c**) GCA. (**d**) Stress–strain curves of the graphene aerogels [24].

**Figure 4:**
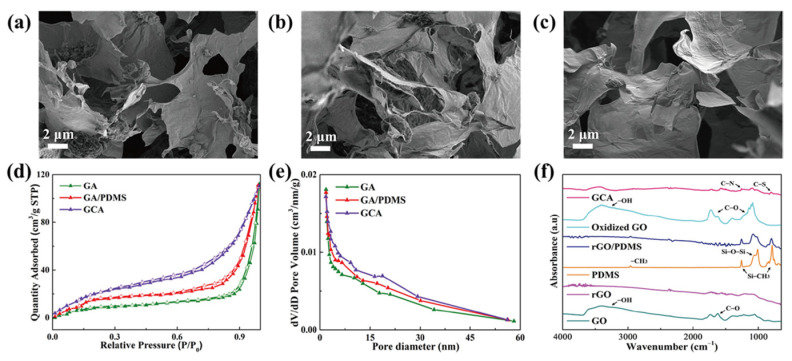
SEM images of (**a**) GA, (**b**) GA/PDMS, and (**c**) GCA. Graphene aerogels BET measurement of (**d**) nitrogen adsorption–desorption peaks, (**e**) pore size distribution peaks, and (**f**) FTIR peaks of the graphene supporting materials.

The authors state that the scientific conclusions are unaffected. This correction was approved by the Academic Editor. The original publication has also been updated.
